# Complete necrosis of hepatocellular carcinoma after preoperative portal vein embolization: a case report

**DOI:** 10.1186/s13256-016-1160-8

**Published:** 2017-01-04

**Authors:** H EL Bacha, M Salihoun, N Kabbaj, A Benkabbou

**Affiliations:** 1 Faculté de médecine et de pharmacie de Rabat, Mohammed V University, Rabat, Morocco; 2 Explorations Fonctionnelles Digestives, Ibn Sina Hospital, Rabat, Morocco; 3 Surgical Department A, Ibn Sina Hospital, Rabat, Morocco

**Keywords:** Hepatocellular carcinoma, Portal vein embolization, Liver resection, Complete tumor necrosis

## Abstract

**Background:**

Hepatocellular carcinoma has a poor prognosis; few patients can undergo surgical curative treatment according to Barcelona Clinic Liver Cancer guidelines. Progress in surgical techniques has led to operations for more patients outside these guidelines. Our case shows a patient with intermediate stage hepatocellular carcinoma presenting a good outcome after curative treatment.

**Case presentation:**

We report the case of an 80-year-old Moroccan man, who was positive for hepatitis c virus, presenting an intermediate stage hepatocellular carcinoma (three lesions between 20 and 60 mm). He presented a complete tumor necrosis after portal vein embolization and achieved 24-month disease-free survival after surgery.

**Conclusions:**

Perioperative care in liver surgery and multidisciplinary discussion can help to extend indications for liver resection for hepatocellular carcinoma outside European Association for the Study of the Liver/American Association for the Study of Liver Diseases recommendations and offer a curative approach to selected patients with intermediate and advanced stage hepatocellular carcinoma.

## Background

Less than one out of three patients diagnosed with hepatocellular carcinoma (HCC) may undergo a curative treatment: liver resection, liver transplantation, or percutaneous ablation [1]. Refinement of surgical techniques and perioperative care helped to extend indications for liver resection for HCC outside European Association for the Study of the Liver (EASL)/American Association for the Study of Liver Diseases (AASLD) recommendations with encouraging short-term and long-term outcomes [2]. When a liver resection of more than three segments is considered, preoperative portal vein embolization (PVE) is of critical importance because it induce a significant growth of the remnant liver, and prevent a potentially lethal postoperative liver failure [3]. Because of a main arterial supply, HCC may respond to transarterial embolization or transarterial chemoembolization (TACE) but not PVE. We report a case of a complete histological necrosis of a HCC after preoperative right PVE.

## Case presentation

**Fig. 1:**
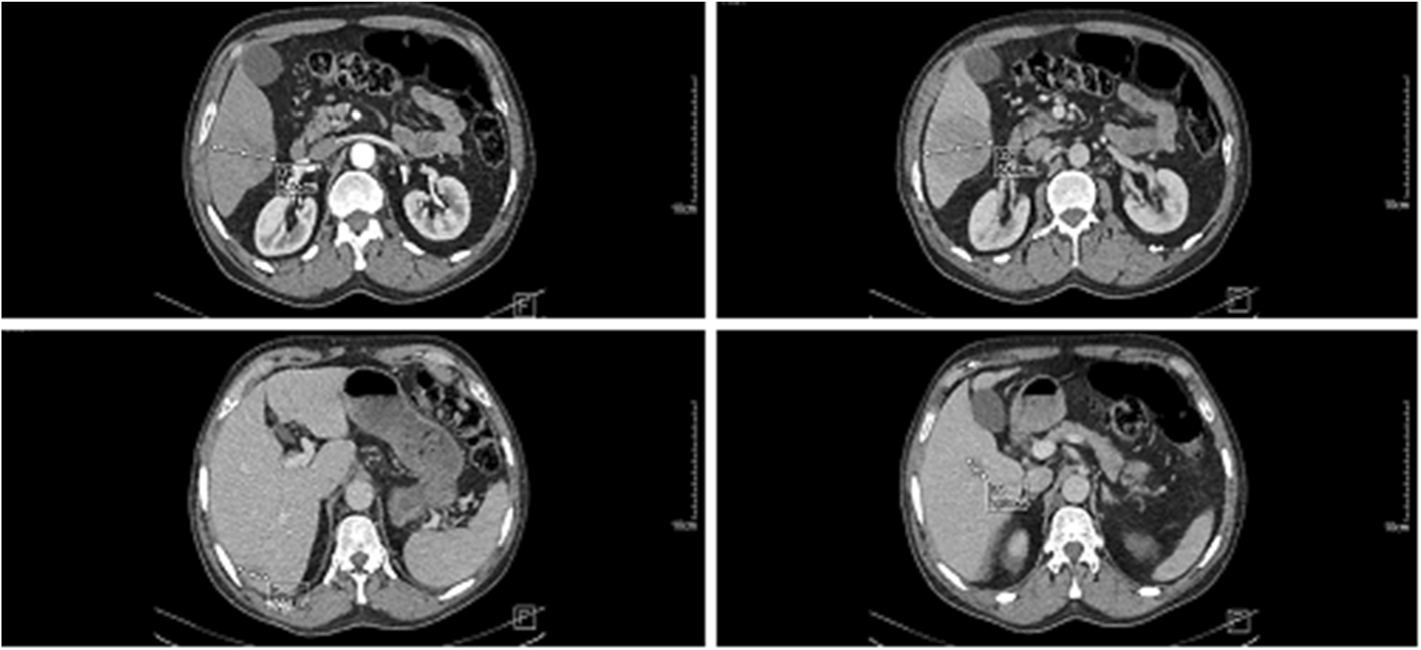
Enhanced computed tomography images showing three liver lesions of 20 mm (liver segment 7), 27 mm (liver segment 5), and 60 mm (liver segment 6)

An 80-year-old Moroccan man presented with a 4-month history of nonspecific abdominal pain and asthenia, he had no comorbidity except late onset asthma. His performance status was good (PS 0 to 1) and a physical examination unremarkable. Abdominal imaging (ultrasound and contrast-enhanced computed tomography) showed three liver lesions of 20 mm (liver segment 7), 27 mm (liver segment 5), and 60 mm (liver segment 6). None of the three lesions fulfilled imaging diagnosis criteria for HCC (Fig. 1; poor arterial enhancement). His spleen size was normal and there was no sign of portal hypertension. Laboratory tests found a slight cytolysis of 1.4-fold normal value. His serum dosage of bilirubin and albumin, and prothrombin time were normal. His serum alpha-fetoprotein (AFP) value was 341.40 ng/ml (normal value <5 ng/ml). Hepatitis C virus (HCV) serology was positive for a genotype 1b virus with a viral load of 4.36 log. Hepatitis B virus (HBV) serology was negative. Pathology analysis of percutaneous liver and tumor (liver segment 6) biopsy showed respectively a chronic active hepatitis A3F2 according to METAVIR score and a well-differentiated HCC, staging our patient as an intermediate stage B according to the Barcelona Clinic Liver Cancer (BCLC) algorithm. After a multidisciplinary meeting, we decided his treatment should be a right hepatectomy. Calculation of the future remnant liver volume (FRL) by software technique found FRL (left liver and segment 1) of 340 ml. A percutaneous portal embolization using ipsilateral technique (embolization of right portal vein collaterals through right liver puncture) was performed with subsequent FRL hypertrophy: 640 ml (+53 %) after 4 weeks. An open right hepatectomy was performed. Specimen pathology analysis found three well-limited nodules with necrotic-hemorrhagic remodeling with a total tumor necrosis and lack of perennial tumor cell in microscopy. His postoperative courses were uneventful. He is alive and free from disease after a follow-up of 24 months.

## Discussion

The effect of PVE on HCC remains controversial and is not well established. Some authors have suggested an anti-tumor effect of PVE on HCC related to partial tumor necrosis after portal thrombosis [4] or total tumor necrosis after PVE [5]. Others suggested a pro-tumor effect of PVE on primary tumors of the liver [6, 7] that may be explained by: (1) the compensatory augmentation of the arterial flow to the tumor after PVE [8], (2) expression of cytoprotective genes involved in remodeling and cell repair as a response to the stress, and (3) induction and modulation of cytokine secretion and growth factors such as tumor necrosis factor (TNF) and hepatocyte growth factor (HGF) [7].

Our report showed a complete histological necrosis of a well-differentiated HCC after preoperative right PVE. This result was unexpected because HCC have a predominant arterial vascularization [9]. Hypothesis 1: some HCC may have predominant portal vascularization. In our patient, this may have been the case as initial imaging showed poor arterial enhancement. Hypothesis 2: a spontaneous regression may occur. Spontaneous regression is defined by a complete or partial clearance of malignant cells in the absence of all treatment or in the presence of therapy that is considered inadequate to exert a significant influence on the neoplastic disease. From a physiopathology point of view, this phenomenon may be caused by hypoxia due to rapid tumor growth, tumor infarction following hemorrhagic shock, circulatory failure by arterial thrombosis or venous thrombosis, or reactivation and recognition of tumor cells by the immune system. Spontaneous regression may occur in approximately 1/100,000 patients with malignant tumor including HCC. Only 12 pathologically proven cases of HCC regression (including our case) have been reported in the literature [10]. Hypothesis 3: an arterial trauma may cause ischemia. In fact, an asymptomatic proximal intrahepatic arterial trauma may be caused during fine liver biopsy and/or percutaneous PVE procedure. This last hypothesis is validated by the facts that in our patient, PVE was performed with multiple right liver (ipsilateral) punctures and that complete necrosis was recorded in all of the three lesions. In our opinion, combined disruption of a predominant portal flow by PVE (hypothesis 1) and arterial flow by a puncture trauma may explain the result in our patient.

Complete necrosis should have been suspected while performing a second preoperative liver imaging to assess future liver remnant hypertrophy but it would not have changed our surgical strategy. In fact, relapse of spontaneously necrotized HCC has been reported [11] and viable persistence of tumoral cells has also been often observed despite a diagnosis of advanced tumor necrosis in imaging examinations [12].

## Conclusions

Refinement of perioperative care in liver surgery, including preoperative portal embolization and multidisciplinary discussion, has helped to extend indications for liver resection for HCC outside EASL/AASLD recommendations and offers a curative approach to selected patients with intermediate (our patient) and advanced stage HCC.

## Abbreviations

AASLD: American Association for the Study of Liver Diseases
AFP: Alpha-fetoprotein
BCLC: Barcelona Clinic Liver Cancer
EASL: European Association for the Study of the Liver
FRL: Future remnant liver volume
HBV: Hepatitis B virus
HCC: Hepatocellular carcinoma
HCV: Hepatitis C virus
HGF: Hepatocyte growth factor
PS: Performance status
PVE: Portal vein embolization
TACE: Transarterial chemoembolization
TNF: Tumor necrosis factor
